# Interleukin-4 Responsive Dendritic Cells Are Dispensable to Host Resistance Against *Leishmania mexicana* Infection

**DOI:** 10.3389/fimmu.2021.759021

**Published:** 2022-01-28

**Authors:** Bernard Ong’ondo Osero, Zama Cele, Raphael Taiwo Aruleba, Rebeng A. Maine, Mumin Ozturk, Manfred B. Lutz, Frank Brombacher, Ramona Hurdayal

**Affiliations:** ^1^ Division of Immunology, Department of Pathology, Faculty of Health Sciences, Institute of Infectious Diseases and Molecular Medicine (IDM), South African Medical Research Council (SAMRC) on Immunology of Infectious Diseases, University of Cape Town, Cape Town, South Africa; ^2^ International Centre for Genetic Engineering and Biotechnology, Cape Town Component, Cape Town, South Africa; ^3^ Faculty of Health Sciences, Wellcome Centre for Infectious Diseases Research in Africa (CIDRI), Institute of Infectious Diseases and Molecular Medicine (IDM), University of Cape Town, Cape Town, South Africa; ^4^ Centre for Biotechnology Research and Development, Kenya Medical Research Institute, Nairobi, Kenya; ^5^ Department of Molecular and Cell Biology, University of Cape Town, Cape Town, South Africa; ^6^ Institute of Virology and Immunobiology, University of Würzburg, Würzburg, Germany

**Keywords:** Dendritic cells, IL-4Rα, IL-4, *Leishmania mexicana*, mice

## Abstract

IL-4 and IL-13 cytokines have been associated with a non-healing phenotype in murine leishmaniasis in *L. mexicana* -infected BALB/c mice as demonstrated in IL-4^−/−^, IL-13^−/−^ and IL-4Rα^-/-^ global knockout mouse studies. However, it is unclear from the studies which cell-type-specific IL-4/IL-13 signaling mediates protection to *L. mexicana*. Previous studies have ruled out a role for IL-4-mediated protection on CD4^+^ T cells during *L. mexicana* infections. A candidate for this role may be non-lymphocyte cells, particularly DCs, as was previously shown in *L. major* infections, where IL-4 production drives dendritic cell-IL-12 production thereby mediating a type 1 immune response. However, it is unclear if this IL-4-instruction of type 1 immunity also occurs in CL caused by *L. mexicana*, since the outcome of cutaneous leishmaniasis often depends on the infecting *Leishmania* species. Thus, BALB/c mice with cell-specific deletion of the IL-4Rα on CD11c^+^ DCs (CD11c^cre^IL-4Rα^-/lox^) were infected with *L. mexicana* promastigotes in the footpad and the clinical phenotype, humoral and cellular immune responses were investigated, compared to the littermate control. Our results show that CL disease progression in BALB/c mice is independent of IL-4Rα signaling on DCs as CD11c^cre^IL-4Rα^-/lox^ mice had similar footpad lesion progression, parasite loads, humoral responses (IgE, IgG1, IgG 2a/b), and IFN-γ cytokine secretion in comparison to littermate controls. Despite this comparable phenotype, surprisingly, IL-4 production in CD11c^cre^IL-4Rα^-/lox^ mice was significantly increased with an increasing trend of IL-13 when compared to littermate controls. Moreover, the absence of IL-4Rα signaling did not significantly alter the frequency of CD4 and CD8 lymphocytes nor their activation, or memory phenotype compared to littermate controls. However, these populations were significantly increased in CD11c^cre^IL-4Rα^-/lox^ mice due to greater total cell infiltration into the lymph node. A similar trend was observed for B cells whereas the recruitment of myeloid populations (macrophages, DCs, neutrophils, and Mo-DCs) into LN was comparable to littermate IL-4Rα^-/lox^ mice. Interestingly, IL-4Rα-deficient bone marrow-derived dendritic cells (BMDCs), stimulated with LPS or *L. mexicana* promastigotes in presence of IL-4, showed similar levels of IL-12p70 and IL-10 to littermate controls highlighting that IL-4-mediated DC instruction was not impaired in response to *L. mexicana*. Similarly, IL-4 stimulation did not affect the maturation or activation of IL-4Rα-deficient BMDCs during *L. mexicana* infection nor their effector functions in production of nitrite and arginine-derived metabolite (urea). Together, this study suggests that IL-4 Rα signaling on DCs is not key in the regulation of immune-mediated protection in mice against *L. mexicana* infection.

## Introduction

Leishmaniases are classified as neglected tropical diseases (1, 2) that are caused by *Leishmania* spp; a vector-borne intracellular trypanosomatid protozoan parasite. This disease is characterized by a spectrum of clinical manifestations ranging from self-healing skin lesions of localized cutaneous leishmaniasis, mucocutaneous leishmaniasis, diffuse cutaneous leishmaniasis, and visceral leishmaniasis depending on *Leishmania* parasite species and host immune response (3, 4). Between 700,000 to 1 million new cases of leishmaniasis have been reported annually, with approximately 65,000 deaths (5). Additionally, the prevalence of leishmaniasis is estimated to be over 4.13 million globally and about 1.7 billion people are at risk of infection in more than 98 countries worldwide distributed in all continents except Antarctica (6–8). Cutaneous leishmaniasis (CL) mostly and widely occurs in tropical regions of Western and Central Asia, the Mediterranean basin, and America (5).

Several drugs currently in use for the treatment of various forms of leishmaniasis are known to cause side effects and require a long period of hospitalization for intravenous administration (9, 10). Despite sustained efforts, no effective human vaccine is yet available (11–14). A promising alternative is the use of chemoimmunotherapeutic approaches, which have the potential to increase efficacy and decrease the toxicity of antileishmanial drugs thereby extending their lifespan in clinical use (15). Central to this approach is the identification of correlates of immune protection in the host organism during active infection. Since the early ‘90s, the canonical T-helper (Th) cytokines, interleukin (IL)-4 and IL-13, have been infamously regarded as mediators of susceptibility to CL in mice. However, a paradigm change to this archetypal role was reported in 2001, where early administration of IL-4 during CL in BALB/c mice promoted Th1 immune response leading to the control of the disease (16). IL-4 and IL-13 share a common receptor, IL-4Rα, whose effects in CL have been widely studied in *L. major* (17, 18) infection and to some extent during *L. mexicana* infection (19). The phenotypic difference of IL-4^-/-^ and IL-4Rα^-/-^ from wildtype BALB/c mice after infection with *L. mexicana* indicated that gene knockout mice control the infection with characteristic smaller lesions and decreased parasite burden (19, 20) thereby suggesting a role for IL-4 and IL-13 in susceptibility to *L. mexicana*.

To better understand the specific cell subsets in the host that render IL-4Rα^-/-^ mice resistant to CL, recent studies have shown that deficiency of IL-4Rα signaling on CD4^+^ T cells protects mice from *L. major* infections but the protection is sex-dependent in *L. mexicana*-infected BALB/c mice; with controlled infection in female mice, but persistent infection in male mice (21, 22). Further studies in our laboratory have shown that BALB/c mice lacking IL-4Rα signaling in dendritic cells (DCs) were hyper susceptible to cutaneous *L. major* infection in the footpad (17). These mice had upregulated Th2 responses and impaired macrophage killing capacity, pointing towards the critical role of IL-4Rα signaling on DCs for protection against CL caused by *L. major*. Mechanistically, this pointed to early IL-4 signaling instructing DC-derived IL-12 production early after infection, which contributes to the host to control infection. Given that IL-4Rα^-/-^ mice are protected from *L. mexicana* infection, it could be possible that the absence of DC IL-4Rα signaling in IL-4Rα^-/-^ mice infected with *L. mexicana* could be the primary contributor to protection. Thus, it is interesting to investigate the role of DCs in mice with IL-4Rα specific deletion in DCs following *L. mexicana* infection. Moreover, since the differences in the outcome of CL in BALB/c mice have been shown to depend on the infecting *Leishmania* species (23), we sought to investigate the importance of IL-4Rα signaling on DCs in the control of *L. mexicana* infection in BALB/c mice. To this end, we utilized BALB/c mice with a specific deletion of the IL-4Rα on DCs (17). Herein, we describe the clinical phenotype, humoral and cellular responses of BALB/c mice with IL-4Rα deficiency on DCs during experimental footpad infection with *L. mexicana* promastigotes. In summary, we show that CD11c^cre^IL-4Rα^-/lox^ mice exhibited non-healing lesions similar to their littermate controls and unchanged parasite load in the footpad (FP) and lymph nodes (LN). Furthermore, we found no sex-related difference associated with *L. mexicana* infection in the absence of IL-4Rα expression on DCs, in contrast to that shown for CD4^+^ T cells (21). Importantly, similar type 2 and type 1 antibody production were observed between CD11c^cre^IL-4Rα^-/lox^ and littermate IL-4Rα^-/lox^ mice, conveying a sustained susceptible type 2 humoral responses compared to IL-4Rα^-/lox^ mice. Surprisingly, although Th2 cytokine production was upregulated in CD11c^cre^IL-4Rα^-/lox^ mice, this did not translate to heightened susceptibility. There was unchanged infiltration of myeloid cells (cDCs, macrophages, neutrophils, and monocyte-derived DCs) in the draining LN of CD11c^cre^IL-4Rα^-/lox^ and littermate IL-4Rα^-/lox^ mice. Interestingly, we found that IL-4-mediated DC instruction was unaltered in IL-4Rα-deficient bone marrow-derived dendritic cells (BMDCs) compared to IL-4Rα^+^ BMDCs. Accordingly, the loss of IL-4Rα signaling on CD11c^+^ cells did not alter the maturation or activation. nor the levels of IL-12p70, IL-10, nitrite and arginine-derived urea compared to IL-4Rα^+^ BMDCs. These findings, together, suggest that IL-4/IL-13-responsive DCs are dispensable in host resistance against *L. mexicana* infection. Moreover, IL-4-mediated instruction of DCs during infection appears to be pathogen and strain-specific in the context of *L. major* (24) and *L. mexicana*-induced cutaneous leishmaniasis in mice.

## Materials and Methods

### Mice

Six to eight week old, age and sex-matched IL-4Rα^-/-^ (18), CD11c^cre^IL-4Rα^-/lox^ and IL-4Ra^-/lox^ (17) were used for *L. mexicana* challenge experiments. CD11c^cre^IL-4Rα^−/lox^ mice were generated from CD11c^cre^C57/BL6 mice inter-crossed with IL4Rα^lox/lox^ BALB/c mice (25) and homozygous IL-4Rα^−/−^ BALB/c mice and backcrossed to BALB/c background for nine generations. CD11c^cre^IL4Rα^−/lox^ mice have deficiency of IL-4Rα signaling on both DCs and alveolar macrophages (26). Transgene-bearing CD11c^cre^IL4Rα^−/lox^ were identified by PCR genotyping as described previously (17). Hemizygous littermate (IL-4Rα^-/lox^) mice with functional one allele of IL-4Rα were used as controls in all experiments. All mice were bred and housed in a temperature/humidity-controlled room under a 12-hour light/12-hour dark cycle and maintained in cages with pressurized individually ventilated (PIV) cages under specific-pathogen-free conditions in the animal breeding facility of the University of Cape Town (UCT).

### Ethical Statement

This study was performed according to the recommendations of the South African national guidelines and the University of Cape Town on practice for laboratory animal procedures. All experiments were done according to the Institutional Faculty of Health Sciences, Animal Research Ethics Committee (AEC) (AEC number: 019/001).

### *L. mexicana* Parasite and Mice Infection

*L. mexicana*, LV4 parasites harvested from lesions in the FP of infected wild type (WT) BALB/c mice were cultured in M199 media (Invitrogen) supplemented with 10% Fetal calf serum (FCS) (Gibco) and 0.5% penicillin/streptomycin antibiotics (100 U/ml and 100 µg/ml, Gibco respectively). Parasites were left to grow at 27°C for 5-7 days until they fully differentiated into stationary-phase promastigotes (17).

Confluent parasites were washed with phosphate-buffered saline (PBS) by centrifugation, fixed in paraformaldehyde and counted in a Neubauer chamber. Mice were anesthetized with 12% ketamine (Anaket-V; Centaur Labs) and 8% xylazine (Rompun; Bayer). 2× 10^6^ *L. mexicana* promastigotes in a final volume of 50 µl were humanely inoculated subcutaneously in the left hind footpad.

### Measurement of Footpad Lesions

Mice were monitored daily, and lesion sizes of the left hind footpad were measured weekly using a Mitutoyo internal dial caliper gauge (Brütsch, Zu¨rich, Switzerland) and compared to a baseline of 2mm for 8 weeks. Weights of mice were also measured weekly to assess their health status.

### Quantification of Parasite Using Limiting Dilution Assay

At 8 weeks post-infection, mice were euthanized and parasite quantification in the infected footpad and draining popliteal lymph nodes (pLNs) were performed using limiting dilution as previously described (27). Briefly, the homogenates of footpad tissues were prepared and resuspended in M199 media, whereas single lymph node cell suspensions were prepared by mechanical digestion of pLNs and passing through 40µm cell strainers. LN cells were washed through with DMEM supplemented with 10% FCS, 1M HEPES, 50mM β-mercaptoethanol, and penicillin/streptomycin (100 U/ml/100µg/ml, respectively, Gibco) and 200ul of the footpad, lymph node homogenates were added into respective neat wells for each mouse in a 96-well plate containing 100µl of complete DMEM. A serial 1:2 dilution was made for FP homogenates and LN cell suspensions and plates were incubated at 26°C for 7 days after which they were read microscopically to determine the lowest dilution at which parasites were observed (2^n^).

### Analysis of Myeloid and Lymphoid Cells by Flow Cytometry

Single-cell suspension of 1×10^6^ cells from the draining pLNs were stimulated with 200 µg/ml phorbol myristate acetate (PMA) and 1mg/ml ionomycin for 2 hours followed by 4 hours incubation with 2mM monensin. The cells were stained with 50 µl FACs buffer containing 1% rat serum, 1% FC-γ blocker (clone 2.4G2) (homemade), and a cocktail of fluorophore-conjugated antibodies for 30 minutes at 4°C in the dark. Antibodies used included surface extracellular staining markers: Ly6C-PerCPCy5.5 (clone AL-21), CD11b-V450 (clone M1/70), MHCII-AF700 (clone, M5/114), CD11c-APC (clone, HL3), F4/80- PE-Cy7 (clone, BM8), CD44-FITC (clone IM7), CD4-V500 (clone RM4-5), CD3-A700 (clone 500A2), CD62L-V450 (clone MEL-14), CD19-PerCPCy5.5 (clone 1D3), CD8-APC (clone 53-6.7) (BD Biosciences). For intracellular cytokine staining, single-cell suspensions were fixed in 4%paraformaldehyde and permeabilized with 0.5% saponin buffer and stained with IFN-γ-AF700 (clone XMGL2), IL-4-APC (clone 11B11), and IL-13-PE (clone eBio13A) (BD Biosciences). 100,000 events were acquired on a BD Fortessa (BD flow Biosciences). Data were obtained as a percentage of the total cells acquired. The data were analyzed using FlowJo software version 10 (TreeStar). Absolute cell numbers were calculated as the product of the cell percentage and the total number of cells counted by trypan blue exclusion method in the isolated pLN.

### Lymph Node Cell Stimulation and Cytokine Detection

1 × 10^6^ cells resuspended in DMEM supplemented with 10% FCS and penicillin/streptomycin (100 U/ml/100 µg/ml, respectively) were cultured in 48 well plates coated with 20 µg/ml αCD3 or stimulated with 8x10^6^ heat killed *L. mexicana* promastigotes at 37°C with 5% CO_2_ for 72 hrs. Concentrations of IFN-γ, IL-4 (BD Biosciences), IL-13 (R&D Systems) were detected in the supernatants using ELISA as previously described (17).

### Detection of *Leishmania mexicana* Specific -IgG1, IgG2a, IgG2b, and Total IgE

*Leishmania mexicana* specific-IgG1, IgG2a, and IgG2b were detected in the serum of infected mice using 10 µg/ml coating of *L. mexicana* soluble Leishmania antigens (SLA) in coating buffer and detected using AP-conjugated specific antibodies (Southern Biotech) by ELISA, as previously described (28). Total IgE antibody ELISA was detected in the serum of infected mice by capture ELISA using 1 µg/ml of coating IgE mAb and biotinylated rat anti-mouse IgE (Southern Biotech) as previously described (17).

### Bone Marrow Derived DCs

Generation of BMDCs was performed as described by Lutz et al. (29) with slight modifications. Briefly, 2×10^6^ of bone marrow cells, harvested from femurs and tibia of CD11c^cre^IL-4Rα^-/lox^ BALB/c mice and their littermate controls, were differentiated into BMDCs in presence of complete RPMI1640 media (10% FCS, 0.5% of 100 U/ml penicillin/100µg/ml streptomycin antibiotics) supplemented with 50% GM-CSF supernatant (R10) and incubated at 37°C in 5% CO_2_ for 10 days. 5 ml of fresh R10 media were added to the cells at day 3 and 6 of incubation. On day 10, non-adherent cells were gently collected and counted by trypan blue exclusion method (30).

Subsequently, 5×10^5^ cells were seeded into 48 well plates and stimulated with 1 µg/ml of LPS or *L. mexicana* promastigotes at ratio of 10:1 (i.e 5×10^6^ parasite to 5×10^5^ cells) in the presence or absence of [1000 U/ml] recombinant mouse IL-4 (BD Biosciences) at 37°C, 5% CO_2_ for 48 hrs. The samples were gently pipetted to dissociate DC clusters and to promote contact between parasites and DCs. After 48 hrs, culture supernatant was collected for nitrite assay and pelleted cells were used for determination of urea as described by previously (17). Levels of IL-12 and IL-10 were determined, maturation and activation were assessed by flow cytometry using fluorochrome conjugated antibodies.

### Statistical Analysis

Statistical analysis was performed using a one-way ANOVA test with Bonferroni post-test for multiple comparisons, and results are presented as means± standard errors of the means (SEM). Each experimental group was compared to IL-4Rα^-/lox^ littermate controls. Statistical p values of *p < 0.05, **p < 0.01 and ***p< 0.001 were considered significant.

## Results

### Susceptibility to *L. mexicana* in BALB/c Mice Is Independent of IL-4Rα Signaling on CD11c^+^ Cells

To determine the effects of the absence of IL-4Rα on CD11c^+^ cells in the host response following *L. mexicana* infection, CD11c^cre^IL-4Rα^-/lox^, global IL-4Rα^-/-^ and IL-4Rα^-/lox^ (littermate control) BALB/c mice were infected with 2×10^6^ promastigotes in the hind footpad, and footpad swelling was measured weekly for 8 weeks using a vernier caliper.

**Figure 1:**
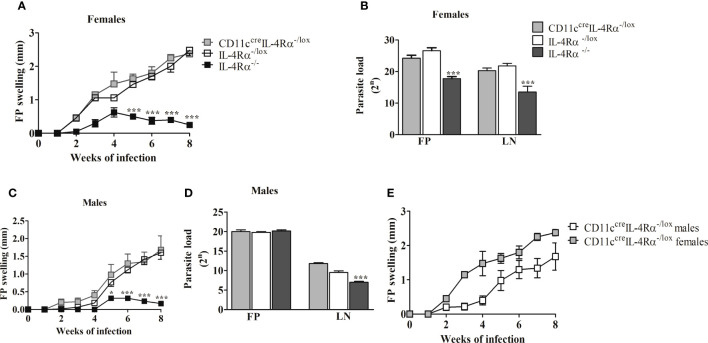
**│**The Footpad (FP) swelling and parasite burden of CD11C^cre^IL-4Rα^-/lox^, IL-4Rα^-/lox^, and IL-4Rα^-/-^ mice subcutaneously infected with *L. mexicana* in the hind footpad. Fp swelling of CD11c^cre^IL-4Rα^-/lox^ and IL-4Rα^-/-^ BALB/c mice were compared to IL-4Rα^-/lox^ control using one-way ANOVA (Bonferroni post-test). FP swelling in female **(A)** and male **(C)**, comparison of Fp swelling between male and female mice **(E)**, and parasite burden in both FP and lymph node (LN) of female **(B)** and male mice **(D)**. Data are presented as the mean ± SEM of 4-6 mice, and P values of ≤ 0.05 were considered significant (*P ≤ 0.05; ***P ≤ 0.001). Data from two experiments are shown.

Similar to previous findings (18, 19) our data show that global IL-4Rα^-/-^ female mice-controlled footpad (FP) swelling in response to *L. mexicana* (**Figure 1A**). In contrast, female CD11c^cre^IL-4Rα^-/lox^ showed fulminant cutaneous leishmaniasis characterized by increasing footpad swelling similar to IL-4Rα^-/lox^ littermate control BALB/c mice during the 8week period (**Figure 1A**). Enumeration of parasites in the footpad and lymph node was performed using a limiting dilution assay (17). Female BALB/c mice deficient for IL-4Rα on CD11c^+^ DCs had similar numbers of parasites in their lesions and pLNs compared to their littermate controls at week 8 post-infection (p.i) (**Figure 1B**). In contrast, IL-4Rα^-/-^ mice had consistently lower parasite burdens in female mice in the FP and dLN compared to littermate control and CD11c^cre^IL-4Rα^-/lox^ mice (**Figure 1B**). Similarly, male CD11c^cre^IL-4Rα^-/lox^ mice displayed non-healing footpad swelling with characteristic systemic parasite dissemination into pLN that was comparable to littermate IL-4Rα^-/lox^ controls (**Figures 1C, D** **)**. Notably, there was no significant difference in kinetics of FP swelling between male and female mice, but a slightly enhanced FP swelling was observed in female mice (**Figure 1E**).

Overall, these findings suggest that the expression of IL-4Rα on CD11c^+^ DCs is dispensable in conferring susceptibility to male and female BALB/c mice infected with *L. mexicana* parasites in the footpad. Given this susceptibility was independent of sex, we focused on the analysis of humoral and cellular responses in female CD11c^cre^IL-4Rα^-/lox^ mice bearing in mind that in previous studies, cell-specific deficiency of IL-4Rα (on CD4^+^ T cells) was particularly relevant in female mice (21).

### The Absence of IL-4Rα Signaling on CD11c^+^ DCs Does Not Alter Humoral Immune Responses in BALB/c Mice Following *L. mexicana* Infection

**Figure 2:**
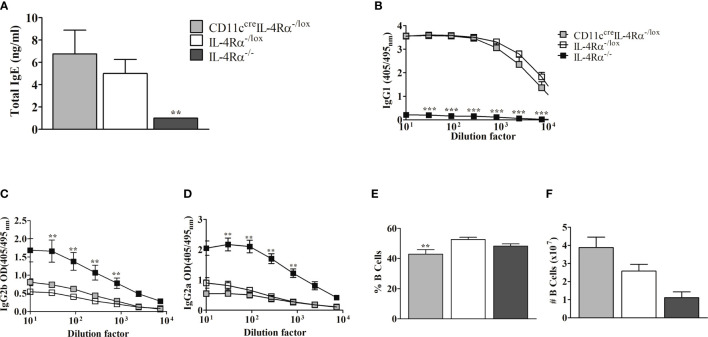
Serum levels of antibody responses and B cell population following infection of CD11c^cre^IL-4Rα^-/lox^, IL-4Rα^-/lox,^ and IL-4Rα^-/-^ BALB/c female mice with *L. mexicana*. Total IgE **(A)** antibodies levels in serum were compared between CD11c^cre^IL-4Rα^-/lox^, IL-4Rα^-/lox,^ and IL-4Rα^-/-^ BALB/c mice. *Leishmania* Ag-specific IgG1 **(B)**, IgG2b **(C)**, and IgG2a **(D)** were measured. The frequency and absolute cell numbers of B lymphocytes were also enumerated from FACs analysis (**E, F** respectively). Data represent means plus SEM values from one experiment representative of two performed is shown. And P values of ≤.01 were considered significant (**P ≤ 0.01; ***P≤ 0.001). Error bars represent the mean ± SE of 4-6 mice per group.

Since CD11c^cre^IL-4Rα^-/lox^ and IL-4Rα^-/lox^ mice displayed non-healing lesion progression (**Figure 1A**), we determined whether the infected mice had a skewed type 2 immune response by first evaluating the levels of type 2-associated IgE and IgG1 (31) in contrast to type 1-associated IgG2a/IgG2b in sera (32). ELISA analysis demonstrated no difference in either total IgE (**Figure 2A**), antigen-specific IgG1 (**Figure 2B**), antigen-specific IgG2b (**Figure 2C** **)** or IgG2a (**Figure 2D**) production between *L. mexicana*-infected CD11c^cre^IL-4Rα^-/lox^ BALB/c mice and littermate controls. In contrast, IL-4Rα^-/-^ mice demonstrated a skewed type 1 humoral response evidenced by reduced IgE and IgG1 (**Figures 2A, B** **)** and concomitantly increased IgG2a and IgG2b (**Figures 2C, D** **)**. These data highlight the correlation between systemic type 2 antibody responses and susceptibility to CL caused by *L. mexicana*. Furthermore, the development of type 2 antibody responses in non-healing cutaneous leishmaniasis occurs independently of IL-4Rα signaling on DCs. An increase in absolute numbers of B cells in both CD11c^cre^IL-4Rα^-/lox^ and IL-4Rα^-/lox^ compared to IL-4Rα^-/-^ mice, thus suggesting that B cells play a role in enhancing the pathogenesis during *L. mexicana* infection (**Figures 2E, F** **)**.

### Type 2 Cytokine Responses Are Increased in CD11c^cre^IL-4Rα^-/lox^ but Did Not Confer Hyper-Susceptibility Over Littermate IL-4Rα^-/lox^ Mice

Susceptibility and resistance to CL in mice are dependent on Th1/Th2 immune responses (33). Therefore, we investigated the role of IL-4Rα signaling on DCs in the cellular response to *L. mexicana* infection by evaluating the cytokine profile (IFN-γ, IL-4, and IL-13) in infected mice by ELISA.

**Figure 3:**
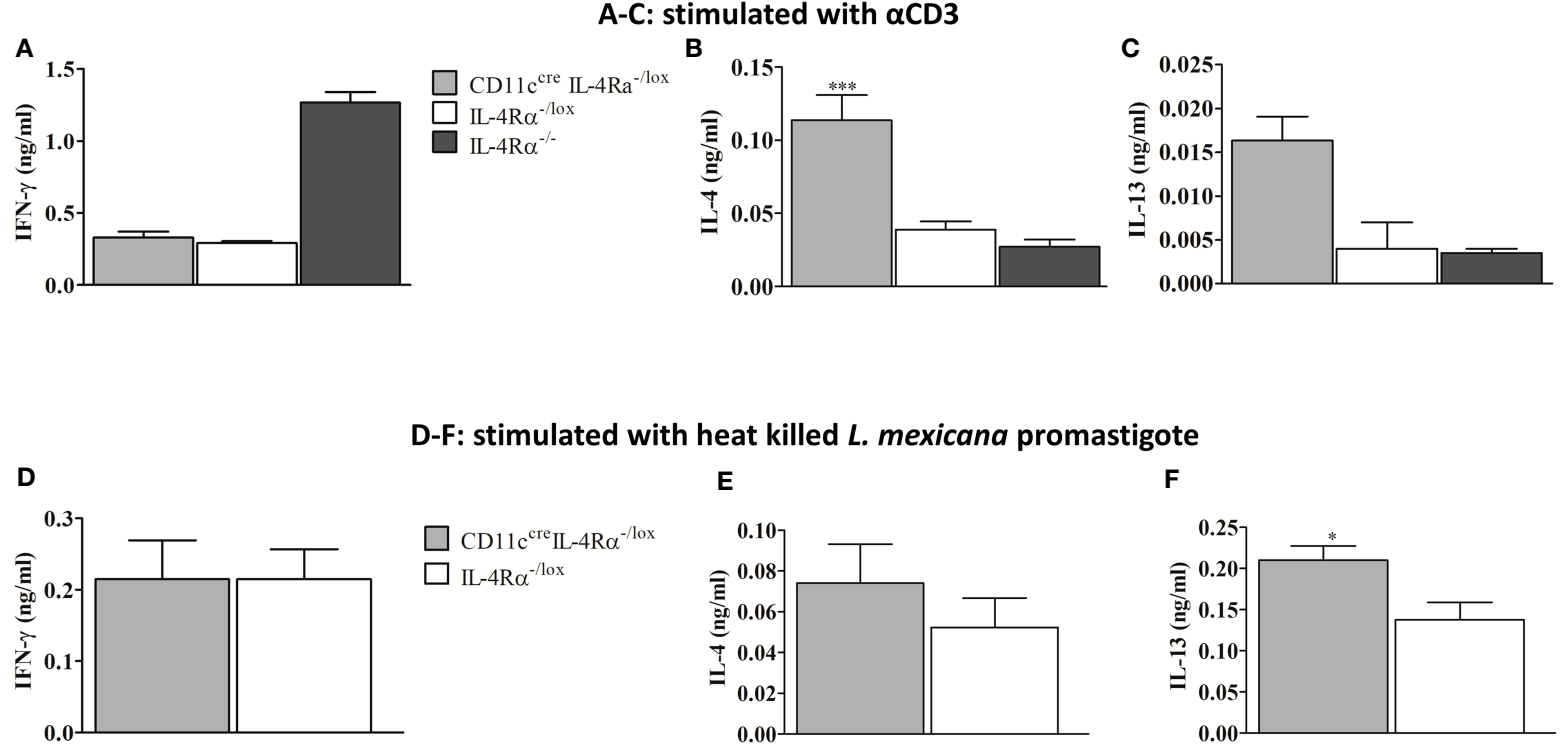
Cytokine response in *L. mexicana*-infected CD11c^cre^IL-4Rα^-/lox^, IL-4Rα^-/lox,^ and IL-4Rα^-/-^ BALB/c mice. Cells from draining Lymph nodes (dLNs) of experimental female mice injected subcutaneously with *L. mexicana* promastigotes at week 8 p.i were stimulated with α-CD3 and heat killed *L. mexicana* promastigotes. IFN-γ, IL-4, and IL-13 cytokines were measured using ELISA. The levels of IFN-γ **(A)**, IL-4 **(B)**, and IL-13 **(C)** secretion by dLN cells stimulated with α-CD3 were shown. Levels of IFN-γ **(D)**, IL-4 **(E)** and IL-13 **(F)** cytokines secretion by cells stimulated with heat killed *L. mexicana* promastigotes was also shown. Data represent means and SEM of values of one experiment representative of two performed is shown. Statistical analysis was performed using one-way ANOVA comparing the differences to IL-4Rα^-/lox^ BALB/c mice as significant (*P ≤ 0.05; ***p = 0.001).

At week 8 p.i the amount of IFN-γ secreted by pLN cells of female mice was similar between CD11c^cre^IL-4Rα^-/lox^ and IL-4Rα^-/lox^ mice but lower compared to IL-4Rα^-/-^ mice (**Figure 3A**). The higher levels of IFN-γ in IL-4Rα^-/-^ mice correlated with the lower FP swelling seen in female mice (**Figure 1A**). Additionally, we investigated whether type 2 cytokines in CD11c^cre^IL-4Rα^-/lox^ and control mice were responsible for susceptibility. On analysis of cytokine from LN cells stimulated with αCD3, we surprisingly found that IL-4 secretion by lymph node cells was significantly increased in CD11c^cre^IL-4Rα^-/lox^ as compared to IL-4Rα^-/lox^ and IL-4Rα^-/-^ mice (**Figure 3B**). Notably, there were also increased IL-13 levels in CD11c^cre^IL-4Rα^-/lox^ as compared to IL-4Rα^-/lox^ and IL-4Rα^-/-^ mice although no statistically significant differences were observed (**Figure 3C**). To supplement the αCD3 stimulated cytokine secretion by LN cells, we restimulated lymph node cells with 8x10^6^ heat killed *L. mexicana* promastigotes in order to investigate the antigen-specific cytokine responses. The data show similar levels of IFN-γ but an increasing trend of IL-4 in CD11c^cre^IL-4Rα^-/lox^ mice compared to their littermate controls (**Figures 3D, E** **)**. In addition, IL-13 levels were significantly increased in CD11c^cre^IL-4Rα^-/lox^ compared to their littermate controls (**Figure 3F**). These findings suggest that IL-4/IL-13 signaling through IL-4Rα on DCs does not influence the production of host-protective cytokines (IFN-γ) during *L. mexicana* infection, however, IL-4Rα deficiency on DCs does enhance IL-4 and IL-13 secretion. Interestingly, Hurdayal et al. (17) reported comparable findings with regard to IL-4 and IL-13 levels, which translated to hypersusceptibility to *L. major* yet surprisingly, in the study herein, this does not translate to hypersusceptibility to *L. mexicana* suggesting that CL caused by different *Leishmania* species leads to different phenotypic outcomes (17).

### Loss of IL-4Rα Signaling on DCs Does Not Change the Expansion and Activation of CD4^+^ and CD8^+^ T Cells During *L. mexicana* Infection

**Figure 4:**
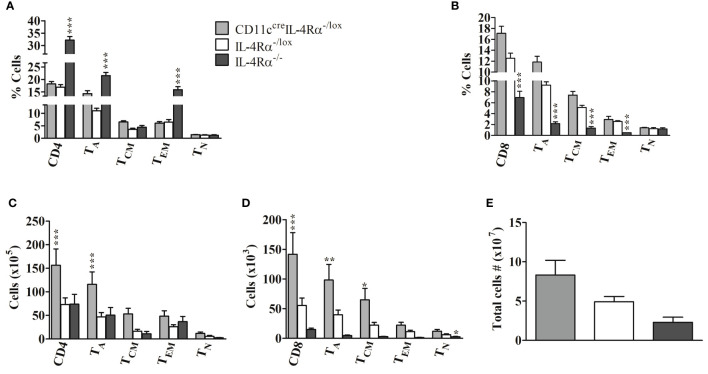
Frequency of lymphocytes during *L. mexicana* infection in CD11c^cre^IL-4Rα^-/lox^, IL-4Rα^-/lox,^ and IL-4Rα^-/-^ mice. Mice were infected subcutaneously with 2×10^6^ *L. mexicana* promastigotes into the hind footpad. At week 8 after infection, frequency of CD4^+^ **(A)** and CD8^+^ **(B)** T cells with their activated (T_A_), central memory (T_CM_), effector memory (T_EM_), and Naïve T cells (T_N_) T cells were enumerated in the LNs by flow cytometry. Absolute numbers of CD4^+^ **(C)** and CD8^+^ **(D)** T cells were calculated and presented. Cells were differentiated based on following markers; CD4^+^ T cells (CD4^+^CD3^+^), CD8^+^ T cells (CD8^+^CD3^+^), T_A_ (CD4^+^CD8^+^CD44^+^), T_CM_ (CD4^+^/CD8^+^CD44^+^CD62L^+^), T_EM _T cells (CD4^+^/CD8^+^CD44^+^CD62L^-^) and T_N_ (CD4^+^/CD8^+^CD44^-^CD62L^+^). Total cell numbers in the LN were also evaluated **(E)**. Data from one experiment representative of two performed are shown. Statistical analysis was performed using one-way ANOVA comparing differences to IL-4Rα^-/lox^ BALB/c mice as significant (*P ≤ 0.05; **p ≤0.01; ***p ≤ 0.001).

Next, we sought to determine if the absence of IL-4Rα^+^ DCs in CD11c^cre^IL-4Rα^-/lox^ mice altered the abundance and involvement of CD4^+^ and CD8^+^ T lymphocytes in the draining pLN following *L. mexicana* infection compared to those of IL-4Rα^-/lox^ mice by flow cytometry analysis (**Supplementary Figure 1**). Overall, lymph node cells comprised a similar percentage of both CD4^+^ and CD8^+^ T cells in CD11c^cre^IL-4Rα^-/lox^ and IL-4Rα^-/lox^ female mice (**Figures 4A, B** **)**. In contrast, we found that the percentage of CD4^+^ T cells in LN of IL-4Rα^-/-^ mice was significantly increased (**Figure 4A** **)** whereas the CD8^+^ T cell percentage was significantly decreased (**Figure 4B**). In terms of effector T cell function, there were no significant differences in CD4^+^ T cell activation (T_A_), effector memory (T_EM_), and central memory (T_CM_) subsets between CD11c^cre^IL-4Rα^-/lox^ mice and their littermate controls (**Figure 4A**). In contrast, activated CD4^+^ T cells and EM in IL-4Rα^-/-^ were showing an increasing trend in CD11c^cre^IL-4Rα^-/lox^ mice whilst T_CM_ levels were similar to IL-4Rα^-/lox^ mice (**Figure 4A**). With regards to CD8^+^ T cells, no differences in frequency of both activated, central, and effector memory CD8^+^ T cells were found in CD11c^cre^IL-4Rα^-/lox^ female mice compared to their littermate controls (**Figure 4B**). Contrary to this, IL-4Rα^-/-^ mice had a significantly decreased percentage of activated, central, and effector memory CD8^+^ T cells in comparison to CD11c^cre^IL-4Rα^-/lox^ and littermate controls (**Figure 4B**). Notably, the percentage of naïve CD4^+^ and CD8^+^ T cells in all three groups was not different in LN during *L. mexicana* infection (**Figures 4A, B** **)** highlighting that loss of IL-4Rα signaling on DCs does not alter the expansion of T cell with a naïve phenotype in the LN during *L. mexicana* infection.

Considering absolute cell numbers in the LNs, CD11c^cre^IL-4Rα^-/lox^ mice generally demonstrated an increase in CD4^+^ and CD8^+^ T cells, activation, and central memory phenotype as compared to littermate controls and IL-4Rα^-/-^ mice (**Figures 4C, D** **)**. The absolute cell number is a product of percentages (obtained from flow cytometry) and total cell numbers infiltrating the draining pLN (enumerated by trypan blue exclusion method). Total cell numbers showed an increased trend in the LN of CD11c^cre^IL-4Rα^-/lox^ mice compared to their littermate controls (**Figure 4E**), hence this could be the reason why absolute numbers of CD4^+^ and CD8^+^ T cells were increased in pLN of CD11c^cre^IL-4Rα^-/lox^ mice. Interestingly, absolute numbers of activated CD8^+^ and memory phenotypes were significantly lower in IL-4Rα^-/-^ compared to CD11c^cre^IL-4Rα^-/lox^ and littermate controls (**Figures 4C, D** **)**. No differences were shown in absolute numbers of CD4^+^ and CD8^+^ T cells with naïve phenotype in all three groups but naïve CD4^+^ and CD8^+^ T cells were consistently reduced in IL-4Rα^-/-^ mice (**Figures 4C, D** **)**. Primarily, no statistical differences in total LN cell numbers were shown between the three groups, however, there was a trend in the increased number total cells infiltrating the popliteal dLN in CD11c^cre^IL-4Rα^-/lox^ compared to IL-4Rα^-/lox^ and IL-4Rα^-/-^ mice (**Figure 4E**).

### Mice Without IL-4Rα Signaling on DCs Display Heightened Levels of Th2-Derived IL-4 and IL-13 Cytokines in Response to *L. mexicana*

Despite the absence of IL-4 signaling on DCs and increased IL-4 and IL-13 secreting potential of pLN cells (**Figures 3B, C** **)**, CD11c^cre^IL-4Rα^-/lox^ mice did not show augmented CL as previously reported during *L. major* infection (17). In that study, hypersusceptibility to *L. major* in CD11c^cre^IL-4Rα^-/lox^ mice was linked particularly to increased IL-4 and IL-13 secretion by CD4^+^ T cells because of impaired IL-4-mediated DC instruction (16, 17). Herein, we, therefore, investigated IL-4 and IL-13 production by CD4^+^ and CD8^+^ T cells in CD11c^cre^IL-4Rα^-/lox^ mice, in an attempt to corroborate increased IL-4/IL-13 in LN cytokine data and explain comparable disease progression observed between CD11c^cre^IL-4Rα^-/lox^ mice and their littermate controls.

**Figure 5:**
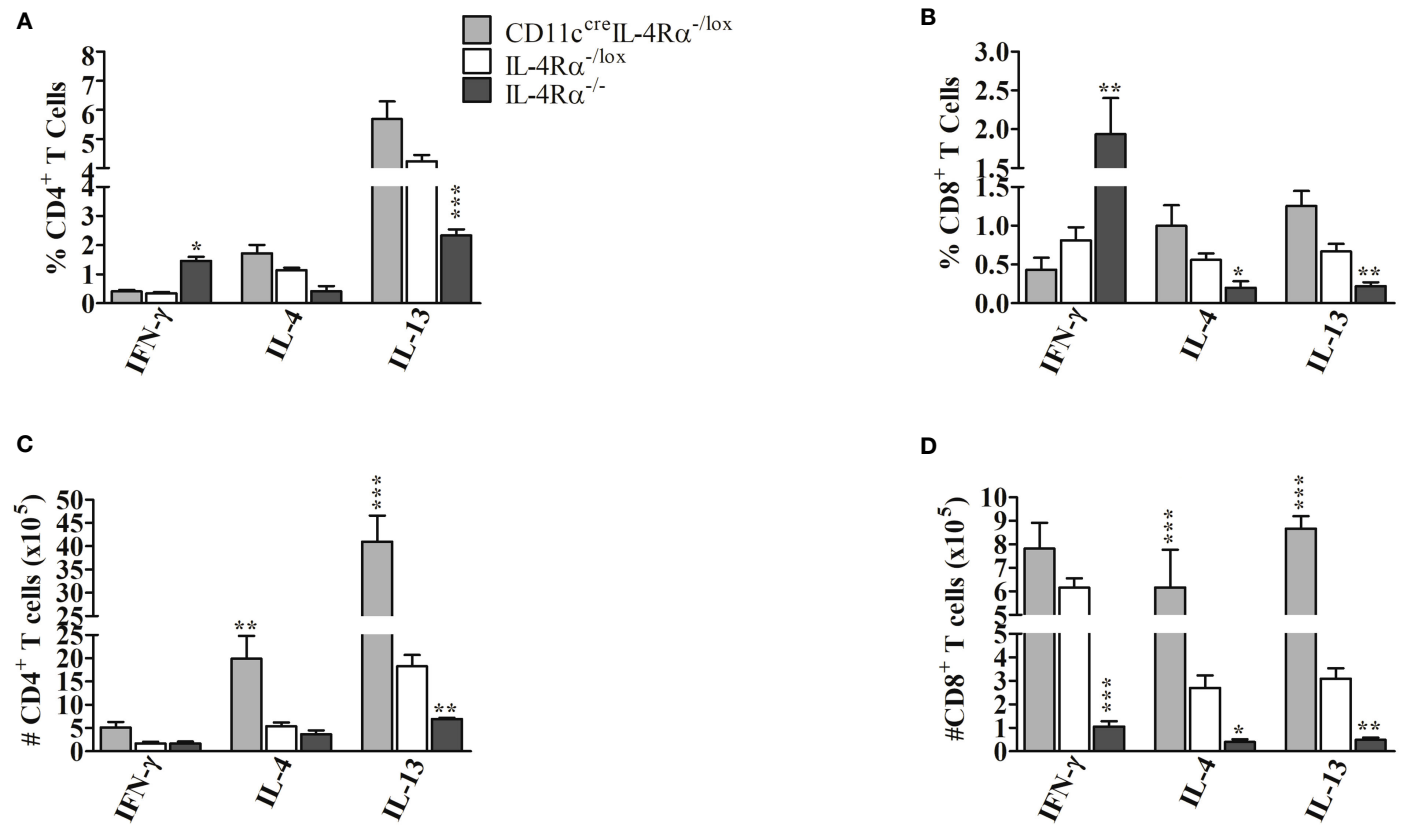
Cytokine producing CD4^+^ and CD8^+^ T cells from CD11c^cre^IL-4Rα^-/lox^, IL-4Rα^-/lox^ and IL-4Rα^-/-^ female mice infected with *L. mexicana*. Female mice were injected subcutaneously in the footpad with 2×10^6^ *L. mexicana* for 8 weeks. 1×10^6^ cells were isolated from popliteal lymph node cells and stimulated with PMA, ionomycin for 2 hours followed by 4hrs incubation with monensin. Percentage CD4^+^ **(A)**, CD8^+^ **(B)**, total CD4^+^ **(C)**, total CD8^+^ **(D)** cytokine-producing T cells were determined by flow cytometry. Statistical analysis was performed using one-way ANOVA comparing the differences to IL-4Rα^-/lox^ BALB/c mice as significant (*p ≤ 0.05; **p ≤ 0.01; ***p ≤ 0.001). Results are from one experiment, a representative of two performed is shown (n=4-6 mice).

In terms of frequency of cytokine-producing cells, our study showed no significant changes in the percentage of CD4^+^ and CD8^+^ T cells producing IFN-γ, IL-4, and IL-13 in both CD11c^cre^IL-4Rα^-/lox^ and IL-4Rα^-/lox^ female mice in contrast to increased IFN-γ, and decreased IL-4 and IL-13 observed in IL-4Rα^-/-^ mice (**Figures 5A, B** **)**. The latter explains the ability of IL-4Rα^-/-^ mice to control infection induced by *L. mexicana* as previously reported (21). Notably, there were increased absolute numbers of CD4^+^ and CD8^+^ T cells secreting IL-4 and IL-13 in CD11c^cre^IL-4Rα^-/lox^ compared to IL-4Rα^-/lox^ (**Figures 5C, D** **)**. We also found that absolute numbers of CD4^+^ T cells producing IFN-γ were comparable amongst the three mouse strains (**Figure 5C**), however, we show significantly decreased CD8^+^ T cells producing IFN-γ in IL-4Rα^-/-^ mice compared to CD11c^cre^IL-4Rα^-/lox^ and IL-4Rα^-/lox^ mice (**Figure 5D**), perhaps due to increased total cell numbers in the pLNs of these mice (**Figure 4E**). Despite the increase in type 2 cytokines by absolute numbers of CD4^+^ and CD8^+^ T cells, the CD11c^cre^IL-4Rα^-/lox^ mice did not show hypersusceptibility as documented previously (17). Potentially, the increased IFN-γ produced by CD4^+^ T cells in CD11c^cre^IL-4Rα^-/lox^ may have been counteracted by the elevated IL-4 and IL-13 (**Figures 5C, D** **)**. Altogether, our findings provide supporting evidence that the absence of IL-4-mediated DC instruction does induce higher levels of IL-4 and IL-13 by CD4^+^ and CD8^+^ T cells and that infection control in this *L. mexicana* setting is dependent on IFN-γ production by T cells which counteracts IL-4 and IL-13 for the survival of the parasites, as observed in the IL-4Rα^-/-^ mice.

### The Deficiency of IL-4Rα Signaling on DCs Does Not Interfere With the Infiltration of Myeloid Cells Into LN During *L. mexicana* Infection

**Figure 6:**
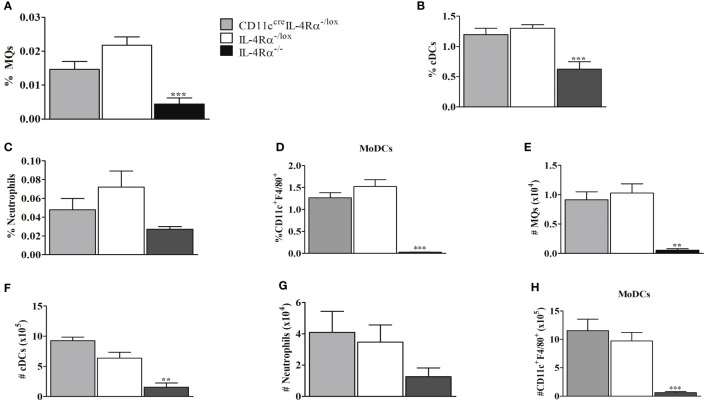
Myeloid cell populations in LNs from CD11c^cre^IL-4Rα^-/lox^, IL-4Rα^-/lox,^ and IL-4Rα^-/-^ mice infected with *L. mexicana*. Mice were injected into the footpad with 2×10^6^ *L. mexicana* metacyclic promastigotes and at 8 weeks post-infection the cells were isolated from LNs and characterized by flow cytometry. The frequency of macrophage **(A)**, conventional DCs (cDC) **(B)**, neutrophils **(C)** and monocyte-derived DCs (Mo-DCs) **(D)** infiltration into LNs were determined. Absolute cell numbers were calculated by multiplying the percentages and total cell numbers in LNs as shown in **(E–H)**. Activated macrophages (MQs) were identified as (CD11b^+^CD11c^-^F4/80^+^MHCII^+^), cDCs (CD11b^+^CD11c^+^MHCII^+^), Neutrophils (Neu) (CD11b^+^L6G^+^), and Mo-DCs (CD11b^+^CD11c^+^F4/80^+^). Statistical analysis was performed using one-way ANOVA comparing the differences to IL-4Rα^-/lox^ BALB/c mice as significant (**P ≤ 0.01; ***p ≤0.001). Results are representative of one of the two experiments.

Since myeloid cells are important for replication and clearance of *Leishmania*, we investigated whether IL-4Rα deficiency on DCs may have modulated myeloid cell recruitment into the LN (24). Infiltration of myeloid cells into LN therefore evaluated for both percentage and total cell numbers using flow cytometry analysis. The cells were identified by gating as shown in **Supplementary Figure 2**. F4/80^+^/CD11c^+^CD11b^+^ can be a typical gate for monocyte-derived DCs (Mo-DCs) with an inflammatory phenotype and monocyte origin, shown to promote CL disease in cell specific DC-IL-4Rα deficient mice (24, 34, 35). CD11c^cre^IL-4Rα^-/lox^ mice had similar percentages of macrophages, cDCs, neutrophils, and Mo-DCs within the LN compared to littermate controls (**Figures 6A–D**) suggesting that the deletion of IL-4Rα in CD11c^+^ DCs did not affect infiltration of these cells in pLN. However, the percentage of macrophages, DCs, neutrophils and monocyte-derived DCs (Mo-DCs) were significantly reduced in global IL-4Rα^-/-^ compared to CD11c^cre^IL-4Rα^-/lox^ mice and their littermate controls (**Figures 6A–D**). Considering the absolute cell numbers, CD11c^cre^IL-4Rα^-/lox^ female mice exhibited an increase in DCs in comparison to their littermate controls whereas macrophages and neutrophil numbers were similar (**Figures 6E–H**). Again, akin to percentages, there was a considerably significant reduction in absolute numbers of macrophages, cDCs, and neutrophils in IL-4Rα^-/-^ mice (**Figures 6E–H**). Altogether, these data indicate that IL-4Rα^+^ expression on CD11c^+^ DCs does not alter the trafficking and expansion of macrophages, cDCs, neutrophils, and Mo-DCs in the draining pLN during *L. mexicana* infection.

### IL-4 Mediated DC Instruction Is Unaltered in CD11c^cre^IL-4Rα^-/lox^ Mice Compared to Their Littermate Controls During *L. mexicana* Infection

**Figure 7:**
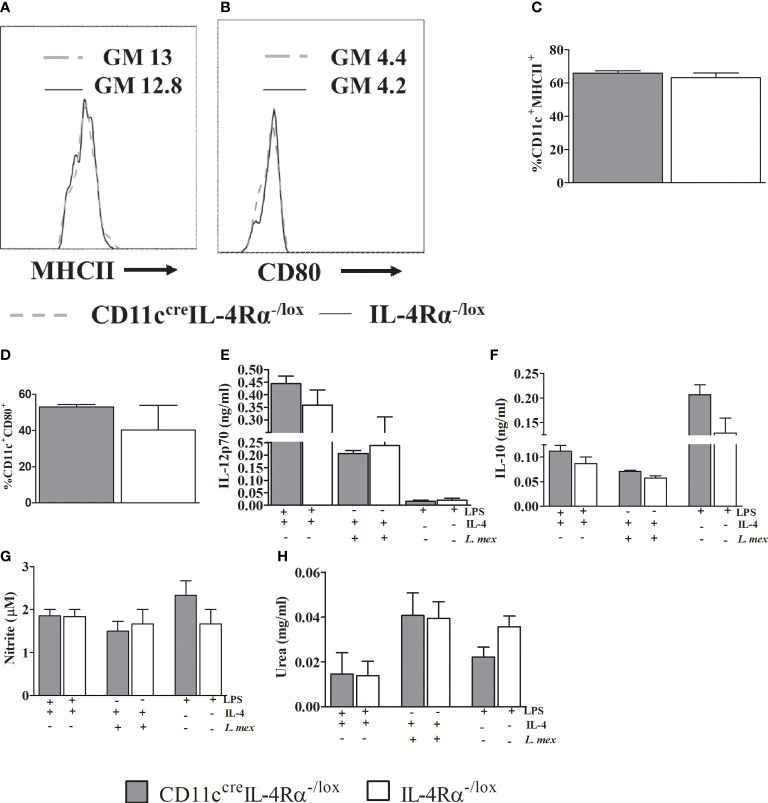
Abrogation of IL-4Rα expression on BMDCs does not either affect instruction or the DC phenotype *in vitro*. Bone marrow cells harvested from the mice were differentiated in RPMI 1640 containing 50% GMCSF supernatant incubated at 37°C, 5 % CO2 for 10 days. 5x10^5^ cells were treated with IL-4 in presence of 1 μg/ml LPS or *L. mexicana* parasites (MOI 10:1). Cells stimulated with LPS only were used as controls. After 48hrs of incubation cells, supernatants were collected for analysis of MHCII **(A, C)**, CD80 **(B, D)**. Histogram showing geometric mean (GM) and % expression of MHCII and CD80 on CD11C^+^ cells is shown. The GM of IL-4Rα^-/lox^ is shown by solid line and CD11C^cre^IL-4Rα^-/lox^ by dashed grey line. IL-12p70 **(E)** and IL-10 **(F)** were measured using ELISA. Supernatants were also used for nitrite assay **(G)** and pelleted cells were used for arginase activity by measuring quantities of urea **(H)**. Data was analysed using one way ANOVA and unpaired student t-test defining differences to IL-4Rα^-/lox^ BMDCs as significant (p ≤ 0.05).

We have shown that CD11c^cre^IL-4Rα^-/lox^ mice had increased IL-4 and IL-13 compared to their littermate controls. IL-4 is known to modulate DC instruction by the production of IL-12 *via* IL-4Rα signaling or impairment of IL-10 (16, 36). To investigate DC instruction in *L. mexicana* infection, we generated BMDCs from CD11c^cre^IL-4Rα^-/lox^ and IL-4Rα^-/lox^ mice. BMDCs were infected and treated with either IL-4 or lipopolysaccharide (LPS) for 48 hours, IL-12p70 and IL-10 cytokine levels were measured in the supernatants. IL-4 stimulation did not alter the maturation and activation of BMDCs as shown by unchanged proportions of MHCII and CD80 respectively, between the two groups (**Figures 7A–D**). Moreover, IL-4Rα -deficient and IL-4Rα sufficient BMDCs produced similar levels of IL-12p70 and IL-10 during infection (**Figures 7E, F** **)** hence suggesting that IL-4-mediated DC instruction was not impaired in CD11c^cre^IL-4Rα^-/lox^.

Nitric oxide (NO) and arginase-1 can be synthesized and secreted by activated DCs during *Leishmania* infection subsequently leading protection or disease severity (17, 37, 38). Since we had found similar numbers of mature DCs in pLN, we wanted to investigate if the mature DCs exhibited any alteration in effector function in their ability to secrete nitrite and urea by virtue of intrinsic iNOS and arginase I activity (39, 40). CD11c^cre^IL-4Rα^-/lox^ BMDCs infected with *L. mexicana* promastigotes and stimulated with IL-4 produced similar levels of nitrite and urea when compared to their littermate controls (**Figures 7G, H** **)**. This unchanged effector function of BMDCs may suggest the similar progressive non-healing lesions and parasite proliferation and dissemination between two strains.

## Discussion

The IL-4Rα pathway, a common receptor for IL-4 and IL-13 signaling, is essential in generating anti-inflammatory responses and mediating susceptibility to infection by *Leishmania* parasites causing CL in mice. In global abrogation of IL-4Rα, the progression of cutaneous leishmaniasis (caused by *L. major* and *L. mexicana*) is severely impaired resulting in protection from infection and eventually a healing outcome (18, 19). Hence there is need for studies on the abrogation of IL-4Rα on specific cells to investigate the cell responsible for resistance to CL infection. IL-4 and IL-13 signaling has been implicated in the maturation and activation of myeloid cells leading to the production of Th1 and Th2 cytokines important for eliminating some parasites (17, 41, 42). In addition, exogenous IL-4 has been shown to instruct DCs to produce IL-12 hence driving healing Th1 immune responses to *L. major* infection in BALB/c mice (16). This was further exemplified *in vivo*, where the abrogation of IL-4Rα on DCs (CD11c^cre^IL-4Rα^-/lox^) led to the host’s susceptibility to *L. major* infection, hence indicating a protective role for IL-4Rα signaling on DCs in BALB/c mice (17). Following this study, we aimed to investigate whether IL-4Rα signaling on CD11c^+^ DCs was the main signaling cascade mediating host protection to *L. mexicana* using CD11c^cre^IL-4Rα^-/lox^ mice and controls. To achieve this, we infected these mice with *L. mexicana* promastigotes in the footpad, and at 8 weeks post infection, we evaluated disease progression, humoral and cellular immune responses.

CD11c^cre^IL-4Rα^-/lox^ mice exhibited non-healing footpad swelling and parasite loads, similar to the littermate IL-4Rα^-/lox^, suggesting that IL-4Rα responsive DCs do not play a role in modulating host-protection to CL disease caused by *L. mexicana*. Sex hormones have been shown to impact the immune responses against leishmaniasis (43). Since, opposite sexes have been found to differ in the intensity, prevalence, and pathogenesis in various infections including CL (44, 45) and in relation to IL-4Rα deletion (21), we compared male and female mice deficient for the IL-4Rα on DCs. By and large, we found no sex-related difference associated with *L. mexicana* infection in the absence of IL-4Rα expression on DCs. It is by this basis we narrowed our investigation to evaluate the humoral and cellular responses in female mice infected with *L. mexicana* parasites bearing in mind that in previous studies, female mice deficient for IL-4Rα on T cells developed a healing phenotype whilst males were unchanged to littermate controls (21).

We next investigated the humoral immune response to explain unaltered disease progression in CD11c^cre^IL-4Rα^-/lox^ and controls. We measured the levels of total IgE and *Leishmania* specific IgG1, IgG2a/b antibodies. The high levels of IgG1 in CD11c^cre^IL-4Rα^-/lox^ and littermate controls compared to IL-4Rα^-/-^ mice may be the causative reason why CD11c^cre^IL-4Rα^-/lox^ mice and their littermate controls were unable to control lesion development. In support, higher titres of IgG1 are present in patients with active CL disease (46). Previous studies built on this evidence and reported that during *L. mexicana* infection, IgG1 preferentially binds to FcγRIII on macrophages thereby suppressing protective immunity, likely through macrophage IL-10 production (47). In addition, other studies have shown that the absence of IgG1 in *L. major* infected mice results in smaller lesions with controlled parasite proliferation (48). Antibody production is dependent on the differentiation of B cells into plasma cells (49). It is therefore not surprising that B cells in totality also do contribute to the host’s susceptibility and are therefore used as correlates of host susceptibility (50). For instance, we show that *L. mexicana* resistant mice (IL-4Rα^-/-^) have decreased population of B cells whilst susceptible CD11c^cre^IL-4Rα^-/lox^ mice and controls have higher numbers. We hypothesize that majority of plasma B cells in CD11c^cre^IL-4Rα^-/lox^ and their littermate controls may be tailored towards IgG1 and IgE antibody production and not protective IgG2a/b antibodies. In summary, our data demonstrate that IL-4Rα signaling on DCs does not lead to events that alter the production of IgG1 and IgE antibodies associated with susceptibility.

IL-4 and IL-13 bind to type I/II receptor that is constitutively expressed on B cells in both mice and humans (51) to induce IgE and IgG1 class switching (52). In our study, higher levels of IL-4 and IL-13 by LN cells stimulated with α-CD3 and heat killed *L. mexicana* promastigotes in CD11c^cre^IL-4Rα^-/lox^ mice did not correlate with heightened levels of IgG1 and IgE compared to littermate controls. Surprisingly, however, the increased Th2 cytokines in CD11c^cre^IL-4Rα^-/lox^ mice did not tilt IgG1 production over littermate controls. The same applies to total IgE, which was significantly lower in IL-4Rα^-/-^ mice but increased in CD11c^cre^IL-4Rα^-/lox^ mice highlighting its systemic role in susceptibility in *L. mexicana* infected mice. Regarding the latter, a clinical study conducted on patients from Brazil and Iraq revealed that high levels of IgE antibodies are associated with disease activity in CL (53, 54). The low levels of type 1 antibodies, namely, IgG2a and IgG2b, exhibited in CD11c^cre^IL-4Rα^-/lox^ and IL-4Rα^-/lox^ mice also further demonstrate the systemic inability of these mice to control the infection as type 1 antibodies have been associated with parasite clearance (17, 32). In this context, a previous study showed that interaction of mouse IgG1-amastigote immune complexes with FcγRIII on macrophages is detrimental in *L. mexicana* infections, but not with interactions involving IgG2a/c (28). Interestingly, IgG2a facilitates the killing of *L. amazonensis* in macrophages (32). Due to the central role of follicular DCs within the germinal center (GC), we speculate that loss of IL-4Rα signaling on DCs did not alter isotype class-switching during GC formation hence no differences in B cell differentiation to plasma cells that could lead to changes in antibody titres (55–57).

The presence of CD4^+^ T cells is important in the control of CL as shown in *L. braziliensis* and *L. major* infection (37). We demonstrated that the loss of IL-4Rα signaling on DCs leads to heightened absolute numbers of CD4^+^ and CD8^+^ T cells. Surprisingly, a negative feedback loop appeared to exist in the activation of T cells and IL-4Rα signaling on CD11c^+^ DCs, with higher levels observed in the absence of IL-4Rα^+^ DCs. Notably, the loss of IL-4Rα signaling on CD4^+^ T cells has been shown to protect BALB/c female mice from *L. mexicana* infection (21).

Central memory T cells (T_CM_), are long-lived cells that can survive without the presence of the parasite and can transform into effector memory cells upon secondary infection (58). Our data reveal an increased absolute T_CM_ cell number in CD11c^cre^IL-4Rα^-/lox^ mice suggesting that these mice have long-term memory hence, they are likely to respond effectively in case of secondary infection with *L. mexicana*. Deletion of IL-4Rα on DCs had no consequences on the infiltration of T cells with naïve phenotype into LNs cells with no prior encounter with *L. mexicana*. Naïve T-cells can differentiate into effector memory T cells (T_EM_) and T_CM_ to facilitate their function in immune responses against infections (59). We also suspect that the interaction between CD4^+^ and CD8^+^ T cells is important in disease control vs susceptibility (60). In support, bystander CD8^+^ T cells can also contribute to a harmful role in CL disease progression (61). On the other hand, in *L. major* infections, CD8^+^ T cells play a vital role in protection (62). In this regard, our results clearly show an increased population of CD8^+^ T cells in LNs of CD11c^cre^IL-4Rα^-/lox^ and IL-4Rα^-/lox^ female mice compared to resistant IL-4Rα^-/-^ mice, suggesting that CD8^+^ T cells perhaps plays a role in the immunopathogenesis of CL caused by *L. mexicana* infection. In an alternate hypothesis, expansion of CD8^+^ T cells in CD11c^cre^IL-4Rα^-/lox^ mice, and their subsequent activation, could lead to degranulation and release of perforin and cytotoxic granzyme effectors, thereby promoting disease severity and mediating immunopathology, as shown in *L. braziliensis*-infected mice (63–65). In parallel, it may be possible that perforin-mediated attack of *L. mexicana*-infected cells prevented hypersusceptibility, contrary to that seen in *L. major* infection (17). Alternatively, this may be either through cell lysis, leading to parasite dissemination, or onset of apoptosis and when apoptotic cells are not cleared by phagocytes, this could lead to an increase in the inflammatory response (66). CD8^+^ T cell effectors generated during *L. mexicana* infection may have tilted to cytotoxic T lymphocytes (CTLs) exhibiting a cytolytic activity and/or lysis of infected cells without killing the parasites hence promoting disease severity, perhaps through granzyme B production, perforins, and IL-1β/inflammasome activation (63, 67). This finding suggests that CD8^+^ T cells may play a minimal role in the protection of the host from *L. mexicana* infections indicating that their presence may result in increased pathology (63). Therefore, an understanding of the mechanisms that drive CD8^+^ T cells to become pathogenic or protective depending on *Leishmania* species, will provide interesting insights.

IFN-γ is unambiguously a host-protector in CL (33). Indeed, studies have shown evidence that IFN-γ secretion by macrophages and DCs fuel a rapid escalation of nitric oxide (NO) production that induces parasite killing (68, 69). Our data show that loss of IL-4Rα signaling on CD11c^+^ DCs does not lead to altered IFN-γ production by CD8^+^ T cells but slight increases in CD4^+^ T cells were observed in the pLN. This may provide an explanation as to why no differences were observed in the footpad lesion progression between CD11c^cre^IL-4Rα^-/lox^ and IL-4Rα^-/lox^ mice even though levels of IL-4 and IL-13 were heightened. Our data, therefore, suggests that the effector functions of IFN-γ on macrophages and DCs remained unchanged by the presence or absence of IL-4Rα signaling on CD11c^+^ DCs. Indeed, it has been documented that the modulation of Type 2 immune responses in murine models of leishmaniasis is more important in the balance of resistance and susceptibility, rather than the Type 1 immune responses. However, the data herein throws caution to this dogma as the heightened IL-4 and IL-13 in CD11c^cre^IL-4Rα^-/lox^ did not equate to hyper-susceptibility as previously reported (17).

Innate cells such as macrophages, neutrophils, and DCs are the first line of defense before adaptive immunity comes into action. Surprisingly, these cells may favor parasite survival hence enhancing disease severity (70, 71). The absence of IL-4Rα signaling on DCs did not affect the infiltration of these innate cells into LN. However, the increased levels in CD11c^cre^IL-4Rα^-/lox^ mice, as compared to resistant IL-4Rα^-/-^, mice suggest that these cells may play a role in susceptibility to *L. mexicana*, perhaps as host cells that favor replication of parasites (71), and thus investigation of their effector responses could be important in controlling CL. The effect of IL-4 in neutrophil counts and migration has been highlighted during bacterial infections (72). This study particularly demonstrated that mice treated with IL-4 have reduced neutrophil migration possibly by affecting CXCR2-CXCR4 chemokine signaling. In our study, we have demonstrated that the absence of IL-4Rα signaling on DCs leads to increased IL-4 cytokines following α-CD3 stimulation, but this did not reduce the infiltration of neutrophils into LN from circulation during *L. mexicana* infection. We thus suspect that the effects of IL-4 on neutrophil migration into the LN may have been counteracted by the presence of the parasite. Transient expression of IL-13Rα2 on DCs may have inhibited IL-4 mediated signaling in littermate controls hence similar myeloid infiltration consequently leading to similar disease phenotype to CD11c^cre^IL-4Rα^-/lox^ (73). It would therefore be interesting to investigate IL-13Rα2 levels in these animals in future studies. Neutrophils may have facilitated the silent entry of parasites into macrophages in a “Trojan Horse” model hence enhancing the infection (74). Considering absolute cell numbers, increased DC population in CD11c^cre^IL-4Rα^-/lox^ mice appears not to reflect in disease control but susceptibility, which suggests that either the loss of IL-4Rα signaling on CD11c^+^ DCs may lead to events that do not cause polarization of these cells to produce pro-inflammatory mediators or signaling in other myeloid cells may be vital in protection against *L. mexicana* infection. More importantly, the loss of IL-4Rα signaling on CD11c^+^ DCs did not affect the production of nitrite and urea (in *ex vivo* infection of BMDCs), which are known substrate for iNOS and arginine-derived metabolite respectively. Specifically, *L. mexicana* infected DCs appear to impair cellular and immunological mechanisms hence leading to the inability to resolve cutaneous disease (39).

Conventional DCs (cDCs) are specialized in uptake of *Leishmania* parasites, process, and present parasite-derived antigens to T cells. More importantly, the cDC1 identified by their CD8α expression as opposed to cDC2, cross-present antigens to CD8^+^ T cells (75, 76). In addition, cDC1 are IL-12p70 producers hence they are less permissive to *Leishmania* infection unlike cDC2 cells that skew CD4 T cell differentiation to Th2 cells (77, 78). Therefore, in our study it is more likely that the cDCs may have majorly been of cDC2 subset, given the increase in IL-4 and IL-13 production by CD4^+^ T cells in the dLN of CD11c^cre^IL-4Rα^-/lox^ mice during *L. mexicana* infection.

Inflammatory DCs, also known as monocyte-derived DCs (mo-DCs) expand during *L. major* infections and been shown to harbour parasites in the lymph node of DC-IL-4Rα deficient mice (24). Analysis on the Mo-DCs revealed unchanged frequencies regardless of the presence or absence of IL-4Rα signaling on DCs. But the IL-4Rα^-/-^ mice that controlled *L. mexicana* infection had decreased frequencies of these cells, demonstrating the vital role they may play in susceptibility to *L. mexicana* infections. In addition, Mo-DCs may have enhanced the differentiation of memory CD8^+^ T cells in both CD11c^cre^IL-4Rα^-/lox^ and their littermate controls compared to global IL-4Rα^-/-^ mice (79).

We also sought to investigate whether the absence of IL-4 signaling in CD11c^cre^IL-4Rα^-/lox^ mice affected IL-4-mediated DC instruction in terms of IL-12p70 and IL-10 cytokine production (16, 36). Surprisingly, the amount of IL-12p70 and IL-10 production by BMDCs was unchanged between the two groups. This observation corroborated with a similar expression of maturation and activation markers such as MHCII and CD80 on CD11c^+^ cells respectively when stimulated with IL-4 (76). Together, we conclude that the loss of IL-4Rα signaling on DCs does not affect their maturation and activation, in addition to unaltered IL-4-DC instruction in IL-12 and IL-10 production. These findings may suggest why we observed similar disease outcome between CD11c^cre^IL-4Rα^-/lox^ mice and their littermates during *L. mexicana* infection.

In summary, our data suggest that IL-4Rα signaling on CD11c^+^ DCs does not have an essential role in the control of CL caused by *L. mexicana*, irrespective of sex and with regard to lack of differences in both humoral and Th1 immune responses. Interestingly, the route or site of infection has been shown to significantly influence the outcome of CL (80, 81), thus it will be interesting to decipher the phenotype exhibited following an intradermal infection with *L. mexicana.* Surprisingly, Type 2 immune responses, in the absence of IL-4Rα signaling on DCs, do increase substantially, yet disease outcome remains similar compared to littermate control. This is paradoxical to some extent because, in the same mouse strain, disease severity is enhanced during *L. major* infection, with a characteristic increase in IL-4 (17). Altogether, this may suggest that the outcome of IL-4-mediated instruction of DCs *in vivo* may be species-specific with respect to CL-causing species.

## Data Availability Statement

The original contributions presented in the study are included in the article/**Supplementary Material**. Further inquiries can be directed to the corresponding authors.

## Ethics Statement

The animal study was reviewed and approved by Faculty of Health Science, Animal Research Ethics Committee, University of Cape Town.

## Author Contributions

Conceptualization: RH and FB. Methodology and investigation: BOO, RA, RH, ZC, RM, MO, and MBL. Writing, Review, and Editing: BOO, MBL, RH, and FB. Funding Acquisition: RH and FB. Supervision: RH and FB. GM-CSF cell line: MBL. Cell-specific gene-deficient mice: FB. All authors contributed to the article and approved the submitted version.

## Funding

This research was supported by funds from the National Research Foundation (grant numbers: 113446 and 120407), University of Cape Town, Research, and Innovation: 2019 Enabling Grant Seeker Excellence Award and Poliomyelitis Research Foundation (grant number: 19/12), under RH. Also, financial support from the National Research Foundation (NRF) of South Africa, Department of Science and Technology (DST), South African Research Chair Initiative, International Center for Genetic Engineering and Biotechnology, and Wellcome CIDRI-Africa (grant number: 203135/Z/16/Z) under FB.

## Conflict of Interest

The authors declare that the research was conducted in the absence of any commercial or financial relationships that could be construed as a potential conflict of interest.

## Publisher’s Note

All claims expressed in this article are solely those of the authors and do not necessarily represent those of their affiliated organizations, or those of the publisher, the editors and the reviewers. Any product that may be evaluated in this article, or claim that may be made by its manufacturer, is not guaranteed or endorsed by the publisher.
