# Supplementary figures and images for: Interleukin-4 Responsive Dendritic Cells Are Dispensable to Host Resistance Against Leishmania mexicana Infection
Source: Front Immunol. 2022 Jan 28;12:759021. doi: 10.3389/fimmu.2021.759021 (PMC8831752; doi:10.3389/fimmu.2021.759021)

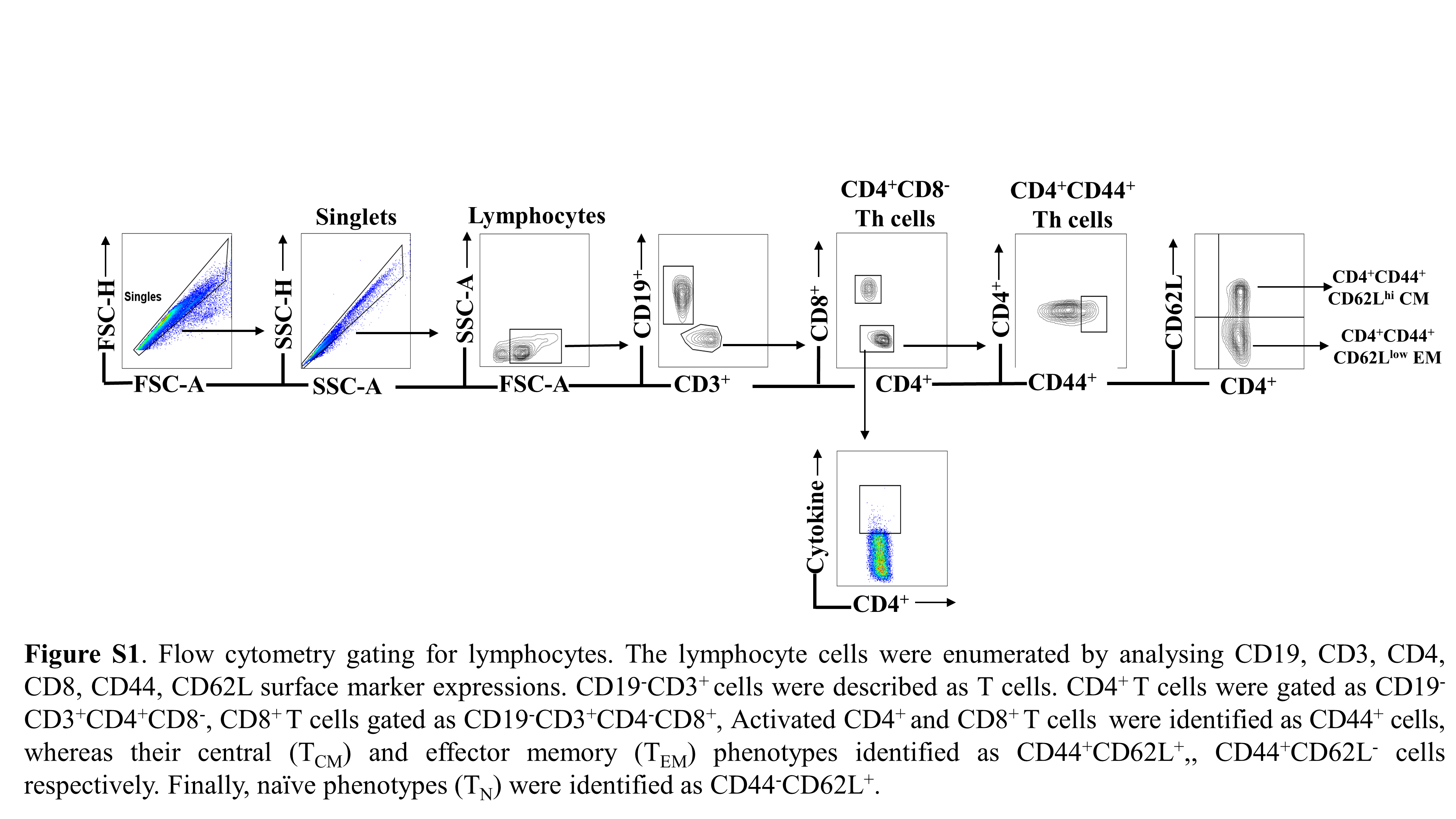

Supplement: Supplementary file 1 [file Image_1.tif]

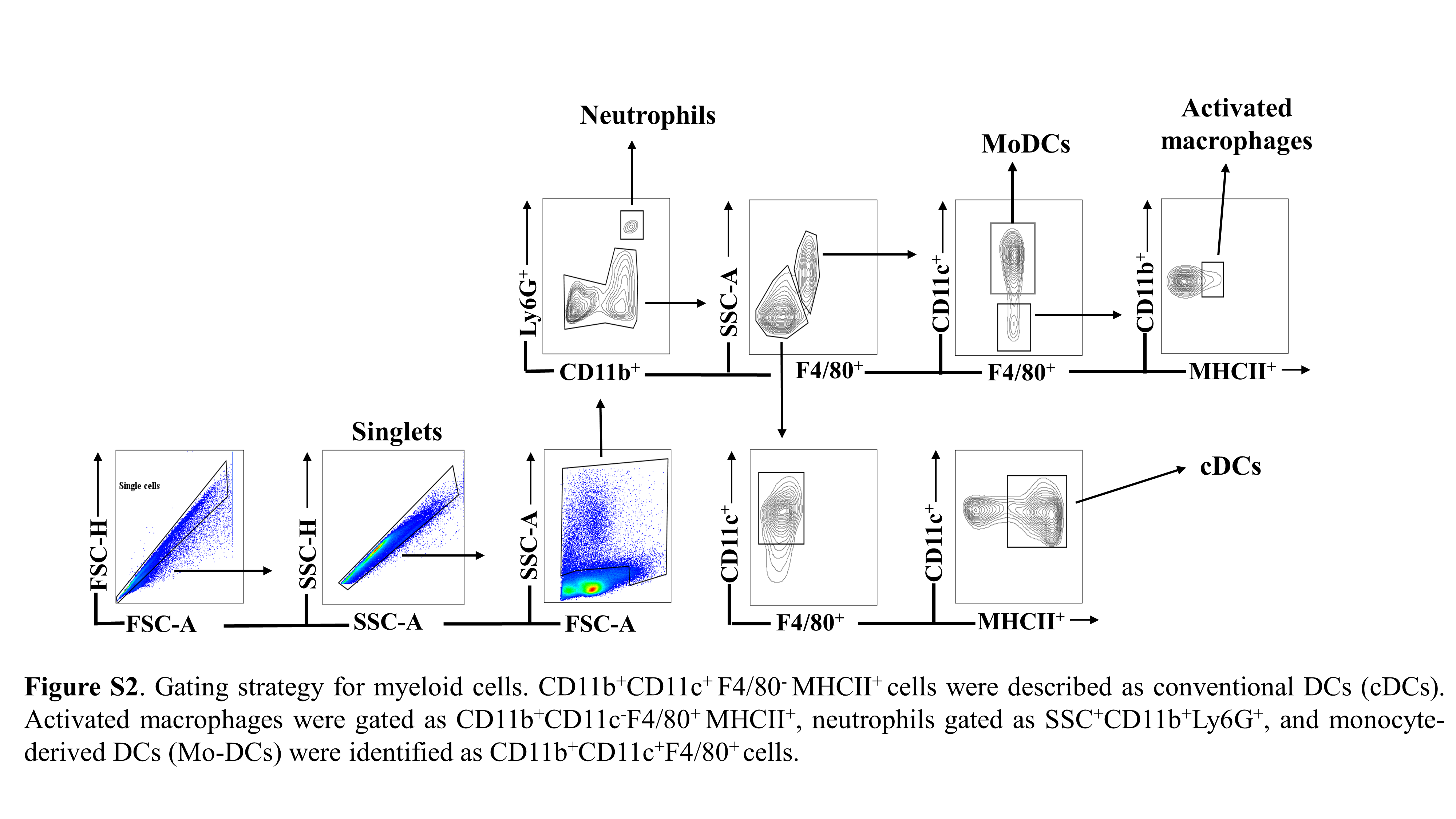

Supplement: Supplementary file 2 [file Image_2.tif]
